# The Interference of *Mnsod3* Enhances the Tolerance of *Pleurotus ostreatus* Mycelia to Abiotic Stress by Reshaping the Cell Wall

**DOI:** 10.3390/jof12010048

**Published:** 2026-01-10

**Authors:** Ludan Hou, Tonglou Li, Baosheng Zhang, Zehua Zhang, Bing Deng, Lijing Xu, Xueran Geng, Yanfen Cheng, Mingchang Chang, Junlong Meng

**Affiliations:** 1 College of Food Science and Engineering, Shanxi Agricultural University, 1 Mingxian South Road, Taigu, Jinzhong 030801, China; 15582690226@163.com (T.L.); 15291884191@163.com (B.Z.); zehuazhang@sxau.edu.cn (Z.Z.); dengbing_edu@163.com (B.D.); xulijingsx@hotmail.com (L.X.); gengxueran2007@163.com (X.G.); cyf2341986@163.com (Y.C.); sxndcmc@163.com (M.C.); 2 Shanxi Key Laboratory of Edible Fungus Germplasm Innovation and Resource Utilization, Taigu, Jinzhong 030801, China; 3 Shanxi Research Center for Engineering Technology of Edible Fungi, Taigu, Jinzhong 030801, China

**Keywords:** *Pleurotus ostreatus*, *Mnsod3*, RNA interference, abiotic stress, cell wall

## Abstract

In recent years, the response mechanism of *Pleurotus ostreatus* to abiotic stress has received widespread attention. MnSOD is an important antioxidant enzyme that has been widely studied in animals and plants because of its functions. However, there is little research on the function and regulatory mechanism of MnSOD in the growth and development of edible fungi. This study investigated the role of *Mnsod3* in the growth and development of *P. ostreatus*. The results showed that during the nutritional growth stage, heat stress can cause the cell wall of mycelia to shrink and the cells to exhibit cytoplasmic wall separation. RNA-seq revealed that *Mnsod3* interference is strongly correlated with increased transcript levels of cell wall synthase genes and with increased tolerance to cell wall disruptors. During the primordium formation stage, the mycelial cell wall also significantly wrinkled under cold and light stresses. RNAi of *Mnsod3* alleviated the cell wall wrinkling caused by cold and light stress, restored the smoothness of the cell walls, and increased mycelial tolerance to abiotic stress. This may be related to the slower formation rate of primordia, but the specific molecular mechanism still needs further research. and slowed the rate of primordium formation. In summary, *Mnsod3* plays an important role in the growth and development of *P. ostreatus* under abiotic stress and plays a critical regulatory role in cell wall remodeling under abiotic stress.

## 1. Introduction

*Pleurotus ostreatus*, which is widely cultivated globally, is highly capable of degrading biomass. In addition, *P. ostreatus* contains abundant active ingredients, such as lovastatin, ergothioneine, melatonin, and other organic compounds, which have potential application value in the healthcare industry [1,2]. However, the cultivation method used for *P. ostreatus* is simple and easily affected by environmental factors, especially during continuous high-temperature weather in summer. At present, the cultivation and management of *P. ostreatus* are rudimentary, resulting in its production often being affected by continuous high-temperature weather, causing the occurrence of “spawn-burning and fungal infection”, which severely affects its quality and yield [3,4]. During the reproductive growth stage of *P. ostreatus*, low temperature and light stimulation are necessary for the formation of primordia. Currently, the physiological basis and molecular mechanism of the response of edible fungi to changes in environmental factors is one of the major scientific issues in China’s edible mushroom industry. However, the regulatory mechanisms of these processes are still unclear. Therefore, the response mechanism of edible fungi to abiotic stress has also become a hot topic for scholars. In recent years, studies on heat stress in *P. ostreatus* have focused mainly on physiological and biochemical changes [3,5], gene function verification [6,7], and signal molecule regulation. For example, recent research has shown that *PoMCA1* positively regulates heat resistance and fruiting body development in *P. ostreatus* mycelia [8]. Nitric oxide (NO), a signaling molecule, can lead to the accumulation of citric acid by inhibiting the activity of aconitase. The accumulation of citric acid further induces the expression of alternating oxidase genes in mitochondria, thereby increasing mycelial heat resistance [9]. Salicylic acid treatment can alleviate mycelial heat stress damage by reducing reactive oxygen species (ROS) levels and increasing the cytoplasmic trehalose content [10]. Several transcription factors that may be involved in regulating mycelial heat tolerance have also been identified. For example, 66 zinc finger transcription factors of *P. ostreatus* were identified and classified into 15 types, 13 of which may be involved in developmental or heat stress responses [11]. In addition, two PoMAC1 transcription factors play opposite roles in the response of mushroom mycelia to heat stress [12].

Superoxide dismutase (SOD) is distributed in plants, animals, and microorganisms. The different active center metals can be divided into three categories: Cu/ZnSOD, FeSOD and MnSOD. Among them, Cu/ZnSODs generally exist in the plant cytoplasm; MnSODs can be found in mitochondria and peroxisomes; and FeSODs are located in chloroplasts and the cytoplasm of plants. In fungi, there are usually both mitochondrial MnSOD and cytoplasmic Cu/ZnSOD. For example, two MnSOD-encoding genes and four Cu/ZnSOD-encoding genes were found in the complete sequenced genome of *Candida albicans*. Three polypeptides with MnSOD activity were detected in the mycelia, zoospores and germinated cysts of *Phytophthora nicotianae* [13]. One Cu/ZnSOD gene and three MnSOD genes were found in *P. ostreatus* [11]. MnSOD, also known as SOD2, is an essential mitochondrial antioxidant enzyme. In animals, the function of SOD2 has been studied most extensively. SOD2 is an essential antioxidant enzyme in mitochondria. Research has shown that in animal models, the absence of SOD2 accelerates mitochondrial dysfunction and promotes cellular aging [14]. Second, SOD2 gene mutations or expression changes are associated with a variety of diseases, including neurodegenerative diseases, cardiovascular diseases, cancer and intervertebral disk degeneration [15]. In plants, MnSOD plays a key regulatory role during growth and development. Research has shown that overexpression of the *Arabidopsis thaliana MnSOD* gene enhances tolerance to oxidative stress during seed germination and early seedling growth [16]. In *Arabidopsis*, *MnSOD* not only regulates the balance of intracellular reactive oxygen species but also participates in regulating root growth [17]. In fungi, *MnSOD* plays a crucial role in maintaining mitochondrial function in response to abiotic stress. For example, in *Schizosaccharomyces*, MnSOD is crucial for the antioxidant stress response and growth of yeast [18]. In *Aspergillus nidulans*, *MnSOD* deficiency increases the sensitivity of mycelia to oxidative stress and promotes cell apoptosis. Moreover, it affects the antioxidant enzyme activity and stress tolerance of conidiospores [19]. At present, there is a lack of in-depth research on MnSOD in large edible and medicinal fungi.

Our preliminary research revealed the presence of three MnSOD-encoding genes in the genome of *P. ostreatus* and investigated the function of *Mnsod1*, which has the highest basic expression level. We found that *Mnsod1* plays a positive role in the growth and development of *P. ostreatus*. In this study, the function of *Mnsod3* in response to heat stress and primordial formation in *P. ostreatus* was studied by constructing overexpression (OE) and RNA interference (RNAi) strains. The changes in the transcription levels of the transformed strains were analyzed through transcriptomics, and the possible regulatory pathways by which *Mnsod3* regulates the growth and development of *P. ostreatus* were explored. On the basis of these findings, the following hypothesis is proposed: *PoMnsod3* serves as a negative regulator of ROS homeostasis in shiitake mushrooms, and its low basal expression level represents an evolutionary fine-tuning mechanism aimed at preventing sustained excessive production of H_2_O_2_. When subjected to abiotic stress, the dynamic regulation of *PoMnsod3* activity reshapes mitochondrial ROS signaling and regulates cell wall remodeling to increase fungal stress tolerance.

## 2. Materials and Methods

### 2.1. Strains and Plasmids

The *P. ostreatus* CCMSSC 00389 strain was provided by the China Center for Mushroom Spawn Standards and Control (CCMSSC). *Agrobacterium tumefaciens* GV3101 (IMCAS, Beijing, China) was preserved in the laboratory and subsequently grown in Luria Bertani (LB) media supplemented with kanamycin and rifampin (Solarbio, Beijing, China). *Escherichia coli* DH5α and BL21(DE3) (TransGen, Beijing, China) strains were used for plasmid construction and protein purification. The prokaryotic expression, OE, and RNAi plasmids were preserved in our laboratory.

### 2.2. Bioinformatics Analysis of the Mnsod3 Gene

The *Mnsod3* gene was identified in previous studies [11]. A phylogenetic tree was constructed via the neighbor connection method in MEGA 5.0 software. The MEME website (https://meme-suite.org/meme/tools/meme, accessed on 10 September 2025) was used for conserved motif analysis of the MnSOD3 protein. The 3D structure of the MnSOD3 protein was predicted via an online website (https://swissmodel.ExPASy.org/interactive, accessed on 10 September 2025). ProtParam (http://web.ExPASy.org/protparam/, accessed on 10 September 2025) was used to predict the molecular weight of MnSOD3. All primers used in this study are shown in Table S1.

### 2.3. Expression and Purification of the MnSOD3 Protein

The expression and purification of the MnSOD3 protein in *Escherichia coli* were performed via our previously described method [20]. The target fragment was ligated into the vector pET28a (Novagen, Inc., Madison, WI, USA) via enzyme digestion and enzyme linkage, and the resulting construct was named pET28a-*Mnsod3*. Afterwards, the pET28a-*Mnsod3* vector was transformed into *E. coli* BL21 (DE3) cells for protein expression. Finally, the expressed MnSOD3 protein was purified by a Ni-NTA column (Qiagen, Duesseldorf, Germany), and the fractions were analyzed by sodium dodecyl sulfate–polyacrylamide gel electrophoresis (SDS–PAGE).

### 2.4. Construction of the OE-Mnsod3 and RNAi-Mnsod3 Plasmids and Strains

At present, OE and RNAi are very effective methods for exploring the gene function of edible mushrooms. In accordance with previously reported methods [6], OE and RNAi plasmids were constructed through homologous recombination, and the exogenous fragments were integrated into the genome of *P. ostreatus* through *A. tumefaciens* GV3101-mediated genetic transformation technology. The transformed strains were subsequently identified and screened via amplification of *hyg* fragments and fluorescence quantification.

### 2.5. Heat Stress Treatment

The wild-type (WT), OE-*Mnsod3*, and RNAi-*Mnsod3* strains were inoculated onto PDA plates. The plates were divided into two groups, with the control group being stored at 28 °C for dark cultivation and the experimental group being stored at 32 °C for heat stress treatment. After 7 days of cultivation, the colony diameter was recorded.

### 2.6. Determination of ROS and Cell Membrane Integrity in Mycelia

The ROS concentration was assessed according to a previously described method, and intracellular ROS production was measured by the fluorescence probe 2′,7′-dichlorodihydrofluorescein diacetate (DCFH-DA) (Beyotime, Shanghai, China) [21]. Two types of fluorescent probes were used for the detection of cell wall integrity. First, the staining solution was prepared by thoroughly mixing 20 µL of fluorescein diacetate (FDA) (Solarbio, Beijing, China), 60 µL of propidium iodide (PI) (Solarbio, Beijing, China), and 920 µL of sterile water. The final concentration of FDA was 100 µg/mL, and the final dose of PI was 60 µg/mL. Afterwards, the mycelia were stained at room temperature for 5 min. The PI-DNA complex had excitation and emission wavelengths of 535 nm and 615 nm, respectively. The excitation wavelength and emission wavelength indicated by the FDA are 488 nm and 530 nm, respectively [22].

### 2.7. Determination of the Growth Rate

The cross method is a traditional and reliable way to measure the growth rate of fungal hyphae. The diameter (mm) of each colony was measured in two perpendicular directions via a Vernier caliper with a resolution of 0.01 mm.

### 2.8. RNA-Seq of Different Strains Under Heat Stress

To further investigate the reasons why RNA mutant strains increase mycelial tolerance to heat stress, mycelial samples from different strains under heat stress were collected for RNA isolation and cDNA synthesis. First, according to the instructions of the reagent kit, total RNA (Invitrogen, Carlsbad, CA, USA) was extracted from different samples. An RNA library preparation kit (New England BioLbs, Ipswich, MA, USA) (NEB) was used to construct the RNA libraries and Illumina NovaSeq 6000 sequencing was performed. The sequencing data are available under the login number SRA501186 in NCBI.

### 2.9. H_2_O_2_ Tolerance Determination

H_2_O_2_ is an important component of ROS. To simulate the increase in ROS in mycelia after heat stress, the tolerance of different strains to ROS was tested by adding H_2_O_2_ to the mycelia. First, PDA culture medium with a final concentration of 5 mmol/L H_2_O_2_ was prepared. Afterwards, the WT, OE-*Mnsod3*, and RNAi-*Mnsod3* strains were inoculated into PDA media supplemented with H_2_O_2_ and incubated at 28 °C for 7 days to observe colony diameter [9].

### 2.10. Fruiting Body Production Experiments with Different Strains

The WT, OE-*Mnsod3* and RNAi-*Mnsod3* strains were activated on potato dextrose agar (PDA) media 3 times. Afterward, according to previous reports [7], mushroom production materials were prepared, with each culture bottle weighing 180 g, having a moisture content of 65%, and sterilized at 121 °C and 0.1 MPa high pressure for 2.5 h for the mushroom production experiments. The different strains were inoculated into cultivation bottles and transferred to a growth room at 25 °C in the dark for cultivation. During the transition from nutritional growth to reproductive growth in *P. ostreatus*, low temperature and light stress are necessary conditions for the formation of primordia. Therefore, the mycelia were placed in cultivation bottles (25 days). The bottles were subsequently transferred to an intelligent mushroom production box. The relative humidity of the air was adjusted to 90–95%, stress with a light intensity of 500 lux for 12 h, and the temperature was maintained at 18 °C. The mixture was then maintained in the dark for 12 h, after which the temperature was set to 10 °C. The primordial formation rates of the different strains were observed, and photos were taken for recording.

### 2.11. Subcellular Localization of MnSOD3

In accordance with our previous methods, *Mnsod3* was cloned, and the pBI121-EGFP-*Mnsod3* vector was obtained through homologous recombination. Afterward, *Agrobacterium tumefaciens* EHA105 was introduced, cultured, collected, and suspended in a concentrated solution (150 mM acetyl eugenol, 10 mM MES monohydrate, and 10 mM magnesium chloride, pH 5.6) to achieve a final OD value of 1.0. Finally, *A. tumefaciens* was injected into tobacco leaves with good growth and cultured for 3 days. The results were observed and recorded via a confocal microscope [12].

### 2.12. Experiments Involving the Addition of Exogenous Cell Wall Antagonists

Two cell wall disruptors, namely, congo red (CR) (Solarbio, Beijing, China) and sodium dodecyl sulfate (SDS) (Solarbio, Beijing, China), were used in this study. PDA plates containing different concentrations of cell wall antagonists (1 mM, 2 mM, 4 mM, 6 mM, and 8 mM) were prepared. The WT, OE, and RNAi-*Mnsod3* strains were cultured on PDA plates and kept in darkness at 28 °C for 5 days. The colony morphology was recorded, and images were taken.

### 2.13. Experiment with the Addition of Exogenous Diethyldithiocarbamate (DDC)

To investigate the role of MnSOD in primitive formation, after the mycelia were filled in the bottles, the culture bottles were evenly divided into different groups before being placed in the intelligent mushroom production box. One milliliter of water was added to the blank group as a control, and 1 mL of 25 mmol/L or 50 mmol/L DDC (Aladdin, Shanghai, China) was added to the experimental group. The rate of primitive formation was observed. Second, to observe the effect of DDC on the growth and development of fruiting bodies, after the formation of primordia, 25 mmol/L or 50 mmol/L DDC was sprayed once a day. The control group was treated with H_2_O. The growth and development of the fruiting bodies were observed. In addition, after the formation of young fruiting bodies, 25 mmol/L or 50 mmol/L DDC was sprayed on the cap, and H_2_O was sprayed on the control group to observe the changes in the cap.

### 2.14. Microscopic Analysis of Mycelia

After the mycelia were subjected to stress treatment, their morphology was examined by scanning electron microscopy (SEM). In accordance with previous methods, mycelial samples were stored in a fixed solution at 4 °C for 24 h. Subsequently, the samples were subjected to three successive washes in phosphate-buffered saline (PBS, pH 7.0), with each wash lasting 15 min. Then, the samples were dehydrated with different concentrations of ethanol, and the samples were placed in a critical point dryer. Finally, the dehydrated samples were coated with gold palladium for 4–5 min and observed via SEM. Changes inside the mycelia under heat stress were observed via transmission electron microscopy (TEM). The mycelia on the PDA plates were cut into small pieces of 1 mm^2^. Then, according to previous methods, the samples were processed and observed with a Hitachi Model H-7800TEM (Tokyo, Japan) [23].

### 2.15. Quantitative Real-Time PCR (qPCR)

Total RNA was extracted from the test sample according to the instructions of the RNA extraction kit (Omega Bio-Tek, Norcross, GA, USA) and reverse transcribed into cDNA with a HiScript II 1st strand cDNA synthesis kit (Vazyme, Nanjing, China). Afterward, gene expression levels were detected via the ChamQ SYBR qPCR master mix kit (Vazyme, Nanjing, China). In accordance with previous research results, *β-actin* was used as the reference gene, and the expression levels of the target genes were calculated via the 2^−△△CT^ method.

## 3. Results

### 3.1. Heat Stress Treatment Causes Membrane Damage and Cell Wall Wrinkling in the Mycelia of P. ostreatus

**Figure 1:**
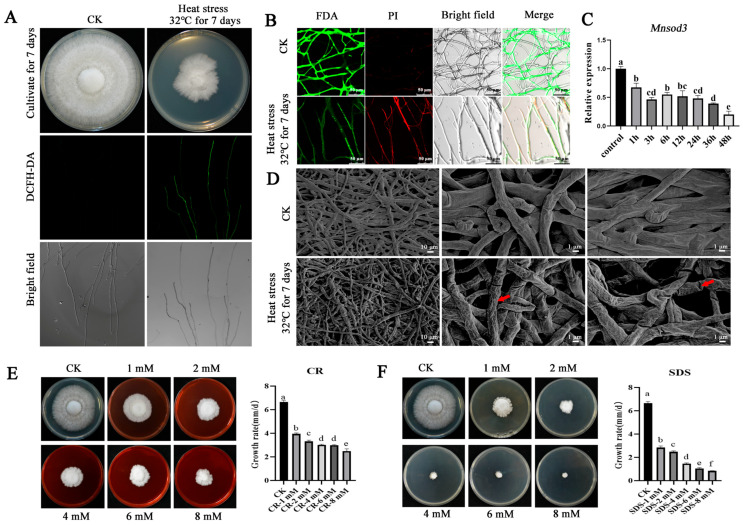
Effects of 32 °C heat stress on the membrane and cell wall of *P. ostreatus* mycelia. (**A**) Heat stress at 32 °C inhibits the mycelial growth rate and causes the accumulation of intracellular ROS. (**B**) The integrity of the mycelial cell membrane is disrupted under 32 °C heat stress. (**C**) Expression of the *Mnsod3* gene after exposure to heat stress for different durations. (**D**) Changes in the mycelial cell wall via SEM. The red arrow points to the cell wall of the mycelium under 32 °C stress. Tolerance of mycelia to different concentrations of CR (**E**) and SDS (**F**). Different letters indicate significant differences among the samples (*p* < 0.05 according to Duncan’s test).

In the cultivation and production of *P. ostreatus*, heat stress is the most critical cause of spawn-burning and fungal infection, ultimately leading to reduced or even complete yield [3,4]. The accumulation of ROS is the fundamental cause of fungal heat stress damage [24]. As shown in Figure 1A, under heat stress, the growth rate of the *P. ostreatus* strain was significantly inhibited, and a large accumulation of intracellular ROS was observed in the mycelia. Further fluorescence probe (FDA and PI) detection revealed that a large amount of red fluorescence appeared in the mycelia after heat stress, indicating that heat stress caused cell membrane damage, resulting in impaired cell membrane integrity and decreased cell activity (Figure 1B). As MnSOD is an important antioxidant enzyme, it plays a crucial role in oxidative stress. Our preliminary research revealed three *Mnsod*-encoding genes in the genome of *P. ostreatus*. Among these genes, *Mnsod1* has the highest basal expression level, and its expression level gradually tends to increase with increasing heat stress duration, indicating that it plays a positive regulatory role in the heat stress response [11]. *Mnsod3* presented the lowest expression level, and its expression level gradually decreased with increasing heat stress duration. It is speculated that *Mnsod3* may play a negative regulatory role in the heat stress response.

The fungal cell wall is a dynamic organelle whose composition strongly affects cell viability and morphogenesis. The walls are built to be both malleable and mechanically robust. Its composition is subject to pressure exerted by the environment [25,26]. Figure 1D shows that when *P. ostreatus* is subjected to heat stress, the mycelia become thinner and the cell wall shrinks. These findings indicate that the cell wall is very important in the response of mycelia to heat stress. The role of the cell wall was further investigated by adding exogenous cell wall disruptors (CR or SDS). Exogenous addition of different concentrations of CR or SDS significantly inhibited the growth rate of mycelia (Figure 1E,F). It can be inferred that the morphological changes in the cell walls were among the important reasons for the significant inhibition of the mycelial growth rate under heat stress.

### 3.2. Bioinformatics Analysis of Mnsod3

In previous studies, we cloned *Mnsod* family genes, including *Mnsod1*, *Mnsod2*, and *Mnsod3*, from the genome of the CCMSSC 00389 strain of *P. ostreatus*. Among the three MnSOD-encoding genes, *Mnsod1* presented the highest basal expression level, whereas *Mnsod3* presented the lowest. In the phylogenetic tree, *Mnsod1* and *Mnsod2* were in one large branch, whereas *Mnsod3* was in another large branch. This study investigated the function of *Mnsod3* during the vegetative growth stage (mycelia) and its potential regulatory pathways during the reproductive growth stage.

**Figure 2:**
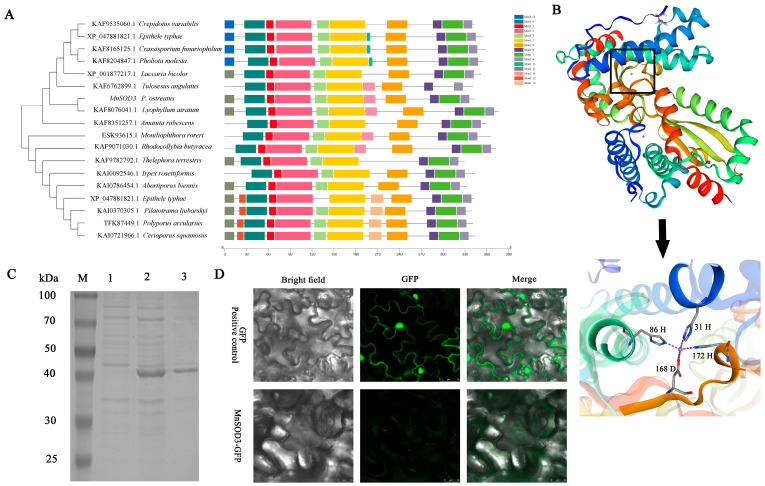
Bioinformatics analysis of MnSOD3. (**A**) Relationships among fungal MnSOD3s. (**B**) 3D structural models. (**C**) Recombinant proteins extracted from *E. coli* BL21 (DE3) cells. M, protein molecular weight standards; 1, crude lysate of MnSOD3 from *E. coli* BL21 (DE3) grown at 16 °C for 12 h; 2, crude enzyme of MnSOD3 from *E. coli* BL21 (DE3) induced with IPTG (1 mM) at 16 °C for 12 h; 3, MnSOD3 protein purified with a nickel column. (**D**) Subcellular localization of MnSOD3.

In previous studies, *Mnsod3* (g12127) was identified in the genome of the *P. ostreatus* CCMSSC 00389 strain, but its biological function has not been studied. In this study, 18 fungal *Mnsod3* sequences were selected to construct a phylogenetic tree. The results indicated that all the MnSOD3 proteins were distributed on two large branches, with MnSOD3 (g12127) having the closest genetic relationship with *Lyophyllum atratum* (KAF8076041.1). The conserved motif results indicated that all the MnSOD3 proteins presented motifs 2, 3, 4, 5, 6, 7, 8, and 9. Moreover, the MnSOD3 sequences of *P. ostreatus* and *L. atratum* had identical motifs, which is consistent with the results of the phylogenetic trees (Figure 2A). The predicted protein tertiary structure results revealed that MnSOD3 is a homologous dimeric protein, and the amino acids at positions 31 (H), 86 (H), 168 (D), and 172 (H) of each monomer interact with Mn metal ions (Figure 2B). The molecular weight of the MnSOD3 protein was predicted to be 38.21 kDa. As shown in Figure 2C, the prokaryotic expression results of the MnSOD3 gene indicated that the actual molecular weight of the MnSOD3 protein was approximately 40 kDa, which is close to the predicted value. The subcellular localization results revealed that GFP signals were present in the nucleus and cytoplasm of the positive control group, whereas the MnSOD3 fusion protein was evenly distributed inside the cell (Figure 2D). Although bioinformatics analysis suggested that MnSOD3 may localize to mitochondria—consistent with most MnSOD family members—the current transient expression assay did not reveal obvious mitochondrial-targeting signals. This discrepancy may arise from differences in the expression system, potential interference from the GFP tag, or species-specific localization patterns.

### 3.3. The Interference of Mnsod3 Increased the Tolerance of Mycelia to H_2_O_2_ and Increased Their Growth Rate Under Heat Stress

**Figure 3:**
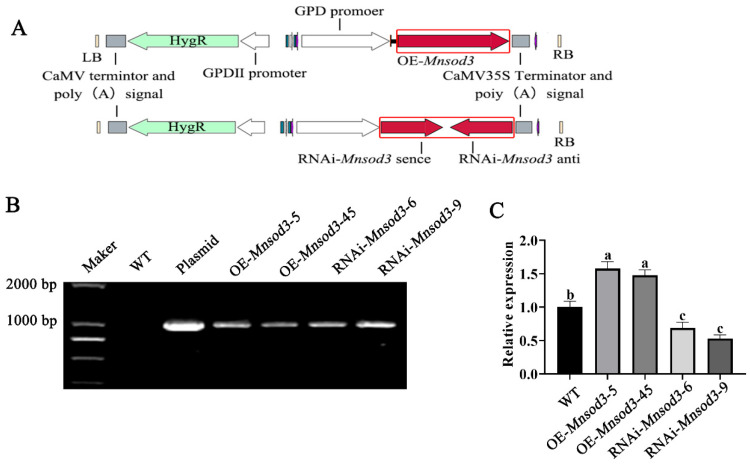
Screening and acquisition of the OE-*Mnsod3* and RNAi-*Mnsod3* strains. (**A**) Plasmid map. (**B**) Amplification of the *hyg* gene in mutant strains. (**C**) Relative expression of *Mnsod3* in the mutant strains. Different letters indicate significant differences among the samples (*p* < 0.05 according to Duncan’s test).

The OE and RNAi plasmid profiles of *Mnsod3* are shown in Figure 3A. Figure 3B shows the amplification of the *hyg* gene fragment in the mutant strain. Figure 3C shows that compared with those in the wild-type (WT) strain, the expression levels of *Mnsod3* in the mutant strains OE-*Mnsod3*-5 and OE-*Mnsod3*-45 were significantly upregulated by 57.56% and 47.83%, respectively. The expression levels of *Mnsod3* in the mutant strains RNAi-*Mnsod3*-6 and RNAi-*Mnsod3*-9 were reduced by 31.19% and 47.02%, respectively.

**Figure 4:**
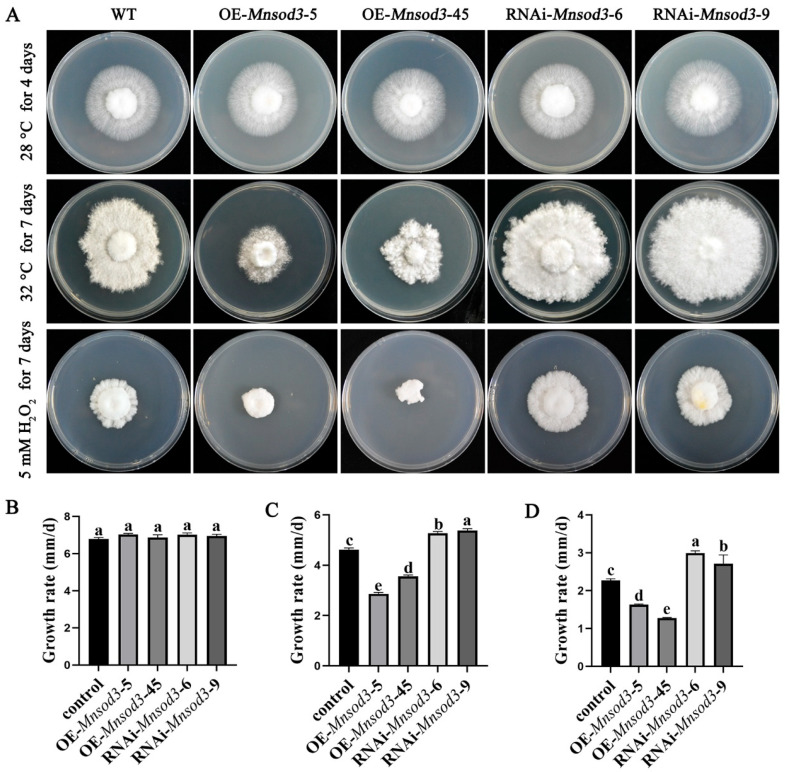
Functions of *Mnsod3* in the vegetative growth stage of *P. ostreatus*. (**A**) Colony morphology of the strains subjected to different treatments. (**B**) Mycelial growth rate at 28 °C. (**C**) Mycelial growth rate under 32 °C heat stress. (**D**) Mycelial growth rate under H_2_O_2_ stress. Different letters indicate significant differences among the samples (*p* < 0.05 according to Duncan’s test).

During the nutritional growth process of *P. ostreatus*, heat stress can cause bursts of intracellular ROS and inhibit mycelial growth. To further investigate the role of MnSOD3 in the response of mycelia to heat stress, the mycelial growth rates of the WT, OE-*Mnsod3*, and RNAi-*Mnsod3* strains were detected under heat stress. The results revealed that under 28 °C cultivation conditions, there was no significant difference in the growth rate of mycelia among the different strains (Figure 4A,B). When the cultivation temperature was increased to 32 °C, compared with those of the WT strain, the colony diameters of the RNAi-*Mnsod3*-6 strain and RNAi-*Mnsod3*-9 strain significantly increased, and the growth rates increased by 13.94% and 16.25%, respectively (Figure 4C). In contrast, compared with those of the WT strain, the mycelial growth rates of the OE-*Mnsod3*-5 and OE-*Mnsod3*-45 strains decreased by 38.10% and 16.25%, respectively (Figure 4C). Under heat stress, a large amount of ROS are produced and accumulate, causing oxidative damage to mycelia. H_2_O_2_ is an important component of ROS. To investigate the tolerance of *Mnsod3* mutant strains to ROS, the tolerance of different strains to exogenous H_2_O_2_ was tested. As shown in Figure 4A, compared with the WT strain, the OE-*Mnsod3*-5 and OE-*Mnsod3*-45 strains only germinated on the pellets after 7 days of cultivation at 28 °C, indicating that the OE of *Mnsod3* enhanced the sensitivity of the mycelia to H_2_O_2_. In contrast, the RNAi of *Mnsod3* increased the strain’s tolerance to H_2_O_2_, resulting in a significant increase in colony diameter compared with that of the WT strain. These findings indicate that *Mnsod3* may play a negative regulatory role in the response to heat stress and that it may participate in the response of mycelia to heat stress by regulating their tolerance to ROS.

### 3.4. RNA-Seq Reveals That Mnsod3 Interference Can Regulate Cell Wall-Related Metabolic Pathways

**Figure 5:**
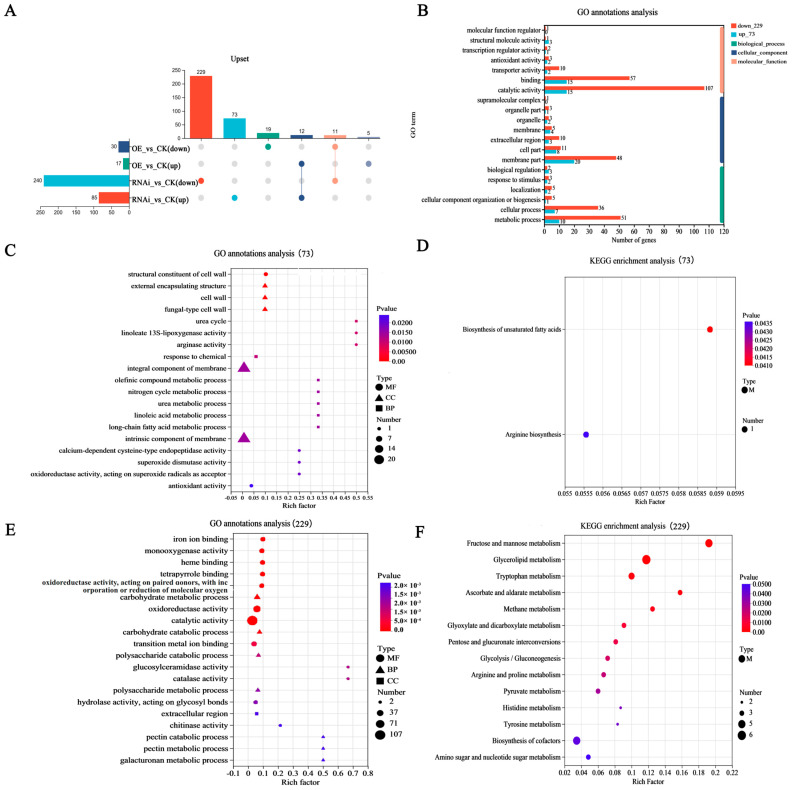
RNAi-*Mnsod3* enhances mycelial tolerance to heat stress by increasing the expression of cell wall-related genes. (**A**) Number of up- and downregulated DEGs among the CK, OE, and RNAi strains. (**B**) GO annotation analysis of the DEGs specifically regulated by the RNAi-*Mnsod3* strains. (**C**,**D**) GO enrichment and KEGG enrichment analysis of DEGs that were specifically upregulated in the RNAi-*Mnsod3* strains. (**E**,**F**) GO enrichment and KEGG enrichment analysis of the downregulated DEGs of the RNAi-*Mnsod3* strains. ).

RNA-seq was used to analyze the changes in gene transcription levels. With a |log2FC| ≥ 2 and *p* value < 0.05, DEGs from different treatment groups were screened and identified. Figure 5A shows that, compared with those in the CK group, a total of 47 DEGs were identified in OE-*Mnsod3*, including 30 upregulated DEGs and 17 downregulated DEGs. A total of 325 DEGs were identified in RNAi-*Mnsod3*, of which 85 DEGs were upregulated and 240 DEGs were downregulated. Further discovery revealed that 302 DEGs can be specifically regulated in RNAi-*Mnsod3*, including 73 upregulated DEGs and 229 downregulated DEGs (Figure 5A). GO annotation analysis revealed that in the biological process category, DEGs were significantly enriched in catalytic activity and binding; in the cellular component category, DEGs were significantly enriched in the membrane part and cell part; and in the molecular function category, DEGs were significantly enriched in metabolic process and cellular process (Figure 5B). The GO functional enrichment results revealed that 73 DEGs that were specifically upregulated in the RNAi-*Mnsod3* strains were significantly enriched in the external encapsulating structure, cell wall, fungal type cell wall, and structural constituent of the cell wall. In addition, 20 DEGs were significantly enriched in the integral component of the membrane and intrinsic component of the membrane (Figure 5C). The 229 DEGs that were specifically downregulated in the RNAi-*Mnsod3* strains were significantly enriched in catalytic activity, oxidoreductase activity, catalase activity, chitinase activity, etc. (Figure 5E). KEGG enrichment analysis revealed that 73 upregulated DEGs were significantly enriched only in the biosynthesis of unsaturated fatty acids and arginine biosynthesis (Figure 5D). The 229 downregulated DEGs were significantly enriched in the fructose and mannose metabolism, glycerolipid metabolism, ascorbic acid and aldehyde metabolism, tryptophan metabolism, and methane metabolism pathways (Figure 5F). Interestingly, our previous research revealed that under heat stress at 32 °C and 36 °C, downregulated DEGs can be significantly enriched in cell wall- and membrane-related pathways, whereas upregulated DEGs can be significantly enriched in oxidoreductase activity, ATP hydrolysis activity, catalase activity, etc. [27]. Therefore, it is speculated that the RNAi of *Mnsod3* may increase mycelial heat tolerance by partially restoring the expression levels of DEGs under heat stress, which may be related to cell wall remodeling and cell membrane structure homeostasis. In addition, several key candidate genes that may play important roles in the stress response are provided in the Supplementary Materials (Table S2).

### 3.5. Interference with Mnsod3 Promotes Cell Wall Synthesis by Positively Regulating the Expression of CHS- and GSC-Encoding Genes

**Figure 6:**
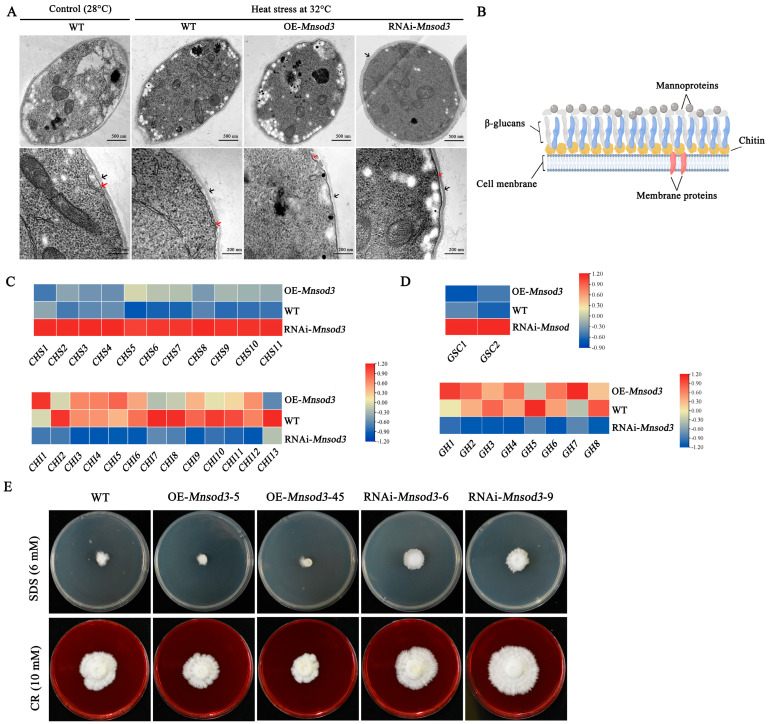
*Mnsod3* negatively regulates cell wall synthesis and enhances mycelial tolerance to heat stress. (**A**) Effects of *Mnsod3* mutation on the mycelial cell membrane and cell wall under heat stress. The red arrow points toward the cell membrane. The black arrow points toward the cell wall. (**B**) Schematic diagram of the cell wall structure. (**C**) Transcriptional levels of genes encoding chitin metabolism-related enzymes (data from RNA-seq). (**D**) Transcriptional levels of genes encoding β-1,3-glucan metabolism-related enzymes (data from RNA-seq). (**E**) Tolerance of *Mnsod3*-transformed strains to SDS or CR.

Mycelial morphological examination after stress revealed that the mycelia in the 32 °C heat stress group separated into cytoplasmic walls compared with those in the control group (28 °C). Under heat stress, compared with the WT strain, the OE-*Mnsod3* strain presented greater cytoplasmic wall separation, and the cell wall became significantly thinner. In contrast, the RNAi of *Mnsod3* weakened the phenomenon of cytoplasmic wall separation caused by heat stress. In addition, the mycelial cell wall thickens, and the color deepens (Figure 6A). These findings indicate that RNAi of *Mnsod3* can promote cell wall synthesis under heat stress.

In recent years, the structure of fungal cell walls has gradually become clearer [24]. Figure 6B shows the fungal cell wall structure, which mainly includes chitin, glucan, and mannoproteins. Figure 6C shows that in the RNAi-*Mnsod3* strain, the expression levels of the chitin synthase-encoding gene family (*chs1-11*) significantly increased compared with those in the WT strain. In contrast, the expression levels of the chitinase-encoding gene family (*chi1-14*) tended to decrease. Figure 6D shows a significant increase in the expression levels of β-1,3-glucan synthase-encoding genes (*gsc1* and *gsc2*) in the RNAi-*Mnsod3* strain compared with those in the WT strain. In contrast, the expression levels of eight genes encoding β-1,3-glucan-degrading enzymes (GH family) were downregulated. The RNA-seq results revealed that the expression of *chs* and *gsc* family genes was significantly upregulated in the RNAi-*Mnsod3* strain, whereas the expression of *chi* and *gh* family genes was significantly inhibited, which is consistent with the phenotype of cell wall thickening and enhanced stress resistance. Although these expression changes are strongly correlated with *Mnsod3* interference, direct or indirect regulation by MnSOD3 remains to be verified via transcriptional network analyses. Furthermore, the tolerance of *Mnsod3* mutant strains to cell wall disruptors was tested. As shown in Figure 6E, compared with that of the WT strain, the growth rate of the OE-*Mnsod3* strains was significantly lower, whereas the growth rate of the RNAi-*Mnsod3* strains was significantly greater.

In summary, RNA-seq revealed that *Mnsod3* interference is strongly correlated with increased transcript levels of cell wall synthase genes and with increased tolerance to cell wall disruptors. Whether this relationship reflects direct transcriptional regulation or an indirect consequence of redox remodeling remains to be elucidated.

### 3.6. RNAi of Mnsod3 Slows the Rate of Primordium Formation by Alleviating Cell Wall Wrinkling

**Figure 7:**
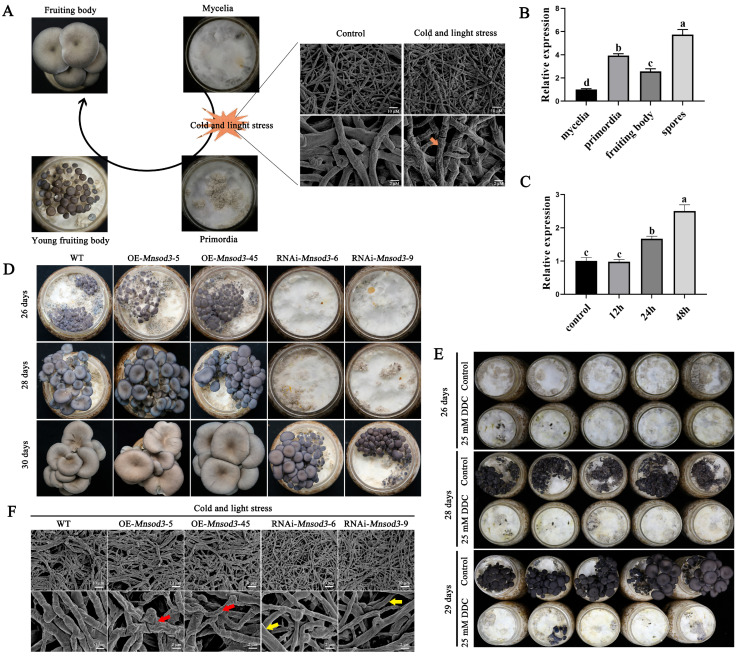
Effects of *Mnsod3* on the rate of *P. ostreatus* primordium formation. (**A**) The impact of cold and light stresses on mycelia during the formation of primordia. (**B**) Relative expression levels of *Mnsod3* at different developmental stages. (**C**) Relative expression levels of *Mnsod3* during the formation of primordia. (**D**) RNAi of *Mnsod3* inhibited the rate of primordium formation. (**E**) The addition of exogenous DDC inhibited the rate of primordium formation. (**F**) RNAi of *Mnsod3* alleviated the cell wall wrinkling caused by cold and light stress. The red arrow points to the cell wall of the OE-*Mnsod3* strains mycelia under cold and light stress. The yellow arrow points to the cell wall of the mycelia of the RNAi-*Mnsod3* strains under cold and light stress. Different letters indicate significant differences among the samples (*p* < 0.05 according to Duncan’s test).

The growth and development of *P. ostreatus* include both vegetative growth and reproductive growth (Figure 7A). Low temperature and light stimulation are essential for the primordium formation of *P. ostreatus*. During the transition from vegetative growth to reproductive growth (the process of primordial formation), the gene expression level of *Mnsod3* gradually increased with increasing light and cold stress duration (Figure 7C). Second, Figure 7B shows that the gene expression level of *Mnsod3* in the primordia increased 2.92-fold compared with that in the mycelia, decreased in the fruiting body, and was highest in the spores, which was 4.74-fold greater than that in the mycelia stage. In summary, *Mnsod3* may play a crucial role in the formation of mushroom primordia. Figure 7D shows that during the growth and development of the fruiting body, there was no difference among the OE-*Mnsod3*-5, OE-*Mnsod3*-45 and WT strains. However, there were significant differences in the rate of primordium formation between the RNAi-*Mnsod3*-6 and RNAi-*Mnsod3*-9 strains and the WT strain (Figure 7D). RNAi of *Mnsod3* inhibited the rapid formation of primordia and prolonged the developmental cycle of fruiting bodies. Second, we aimed to further clarify the influence of *Mnsod3* on the rate of primordial formation. We validated this method by adding the exogenous MnSOD inhibitor DDC [28]. Compared with that in the control group, the rate of primordial formation was significantly lower in the DDC-treated group (Figure 7E). However, DDC can inhibit all metal SODs (MnSOD, Cu/ZnSOD). Therefore, the observed delay in primordium formation cannot be fully attributed to the loss of MnSOD3 function.

To further investigate the mechanisms by which *Mnsod3* affects the formation of primordia, we studied the changes in mycelia of *Mnsod3* mutant strains under cold stress and light stress (conditions required for mushroom production) via SEM. Under cold and light stress, the mycelial cell wall of the WT strain was slightly wrinkled (Figure 7A). In the OE-*Mnsod3*-5 and OE-*Mnsod3*-45 strains, the degree of wrinkling on the mycelial cell wall significantly increased. In contrast, in the RNAi-*Mnsod3*-6 and RNAi-*Mnsod3*-9 strains, the mycelial cell wall was smooth, but mycelial thinning was clearly observed compared with that in the WT and OE-*Mnsod3* strains (Figure 7F).

In summary, during the formation of *P. ostreatus* primordia, the RNAi of *Mnsod3* can increase the tolerance of mycelia to cold and light stresses by alleviating cell wall wrinkling, leading to a slower rate of primordium formation.

## 4. Discussion

SOD is encoded by a small multigene family. Research on MnSOD is extensive and in-depth in animals but very limited in edible mushrooms. To further analyze the function of MnSOD in the growth and development of edible fungi, we identified three MnSOD-encoding genes in the genome of *P. ostreatus* in previous studies, and *Mnsod1* was shown to play a positive role in the response to heat stress and the primordial formation process [11]. This study further investigated the biological function of *Mnsod3*.

The growth and development of *P. ostreatus* can generally be divided into two stages: nutritional growth and reproductive growth. Abiotic stress is necessary for the growth and development of edible mushrooms. Some abiotic stresses can cause mycelial damage and affect yield. Some stresses can promote growth and development, such as light stress and cold stress [29,30]. During the process of nutritional growth, high-temperature stress affects the yield and quality of fruiting bodies. In this study, *Mnsod3* RNAi increased the tolerance of *P. ostreatus* mycelia to heat stress, regulated cell wall-related metabolism, and increased cell wall thickness; in contrast, the OE of the *Mnsod3* strains resulted in reduced tolerance to high temperature. This is completely opposite to the function of *Mnsod1* in *P. ostreatus* [11]. Interestingly, this phenomenon also occurs in other organisms. Many studies have shown that mitochondrial MnSOD plays an important role in aging and lifespan control. In yeast, the absence of *Mnsod*, which encodes mitochondria, was found to decrease the chronological and replicative lifespan [31]. In *Aspergillus nidulans*, the deletion of *Mnsod* significantly enhances the sensitivity of mycelia to antifungal proteins [32]. In mice, the OE of *Mnsod* mitigated anterior cruciate ligament injury-induced muscle atrophy and weakness [33]. Heterozygous mice with one copy of *Mnsod2* are more sensitive to oxidative stress [34]. In addition, a previous study showed that the absence of *Mnsod2* shortened the lifespan of worms and flies [35]. However, few contradictory results have been reported. For example, in yeast, the OE of *Mnsod* prolongs chronological survival, shortens the replicative life span and prevents the budding of some primitive mother cells [36]. In *Caenorhabditis elegans*, the deletion of *Mnsod* extends worm lifespan by altering mitochondrial function [37]. Our preliminary research revealed that *Mnsod1* in *P. ostreatus* plays a positive role in the response of mycelia to heat stress. In contrast, in this study, *Mnsod3* negatively regulated the response of mycelia to heat stress. Previous studies have shown that in *Podospora anserina,* the abundance of mitochondrial SOD (*Pasod3*) is significantly lower in the mitochondrial extracts of senescent fungi than in those of juvenile strains. Further research has shown that the OE of *Pasod3* leads to a shortened lifespan and increased sensitivity to glyphosate and H_2_O_2_ [38]. In this study, the low basal expression of *Mnsod3* may represent an evolutionary adaptation strategy. Its low abundance prevents the sustained production of excessive H_2_O_2_ (SOD catalytic product), thereby preventing excessive interference with cell wall remodeling and energy metabolism. This fine-tuning mode enables hyphae to respond quickly to stress. Similar strategies have also been reported in other species, such as the low expression of *Posod3* in *P*. *anserina*, which is associated with lifespan regulation [38]. Our results are similar to those of previous studies, indicating that some MnSOD-encoding genes play a negative regulatory role in the stress response process in organisms. Therefore, it is speculated that there are significant differences in the number of genes encoded by MnSOD among different species and that there may be significant differences in the functions of different MnSOD-encoding genes within the same species.

The transition from vegetative growth to reproductive growth during the growth and development of edible mushrooms is a hot topic of concern and research by scholars. Multiple functional genes [8,39], transcription factors [40,41,42], and others play important roles in this process [10,43]. The rate of primordial formation is an important indicator of this process [20]. In actual production, the transition from nutritional growth to reproductive growth requires low temperature and light stimulation. Under both types of stress, mycelia entangle and become primordia. Previous studies have shown that mechanical damage can promote an increase in H_2_O_2_ content and shorten the formation time of primordia [44]. Our previous research revealed that H_2_O_2_ gradually accumulates in mycelia during the formation of primordia and that the addition of exogenous H_2_O_2_ can promote the rate of primordium formation [6]. This indicates the importance of H_2_O_2_. This study further revealed that ATP content significantly decreased after the formation of primordia (Figure S1). The leakage of electrons in the respiratory chains is the main cause of ROS production. Therefore, it is speculated that the increase in H_2_O_2_ content is closely related to the decrease in ATP content. SOD can convert superoxide radicals into oxygen (O_2_) and H_2_O_2_ [45]. The OE of *Mnsod3* promotes the rate of primordium formation. This can be achieved by increasing the content of H_2_O_2_. DDC is a SOD inhibitor. This study revealed that the addition of DDC can reduce the rate of primordium formation, which is consistent with the phenotype of the RNAi-*Mnsod3* strains. The addition of DDC can downregulate the transcription levels of *Mnsod1*, *Mnsod2*, and *Mnsod3* during the formation of primordia. The slowing of the rate of primordium formation may be related to the decrease in H_2_O_2_ content. In addition, our research indicates that the addition of DDC plays a crucial role in the growth and development of fruiting bodies (Figure S2). It can downregulate the expression level of the *Mnsod* gene family (Figure S3). Interestingly, the addition of the H_2_O_2_ scavenger DMTU not only reduced the enzyme activity of catalase (CAT) but also increased the ATP content in the primordia. In contrast, the addition of H_2_O_2_ significantly inhibited the ATP content in the primordia (Figure S4). A possible link between redox changes and energy status is suggested. However, direct causation remains to be experimentally established.

The cell wall is also a dynamic organelle whose composition greatly influences the ecology of the fungus and whose composition is highly regulated in response to environmental conditions and imposed stresses [25]. Second, the cell wall is considered the most phenotypically diverse and plastic component of cells [46]. This study revealed that during the growth and development of *P. ostreatus*, regardless of heat stress or cold and light stress, the mycelial cell wall significantly shrinks. RNAi of *Mnsod3* plays a positive role in the abiotic stress response of *P. ostreatus*. Further research revealed that this positive effect is closely related to the cell wall. Previous studies have shown that in fungi, gene expression related to cell wall biogenesis under high-salt conditions is associated with mycelial thickening [47]. Under high-salt conditions, *Aspergillus sydowii* enhances chitin biosynthesis, and the binding of α-glucan results in the formation of thick, hard, and hydrophobic cell walls. This structural rearrangement enables fungi to adapt to high-salt and salt-deficient conditions, providing a powerful mechanism for resisting external pressure [48]. Our research results are similar to theirs. Compared with *Mnsod1* and *Mnsod2*, *P. ostreatus* presented the lowest basic expression level of *Mnsod3.* Moreover, in the RNAi-*Mnsod3* strain, the expression levels of the chitin and β-1,3-glucanase genes, the main components of the cell wall, were increased, whereas the expression levels of the chitinase and glucanase genes were decreased. It is speculated that *Mnsod3* plays a role in the growth and development of *P. ostreatus* in response to abiotic stress and that regulatory effects can be exerted by modulating the synthesis or breakdown of cell walls.

## 5. Conclusions

**Figure 8:**
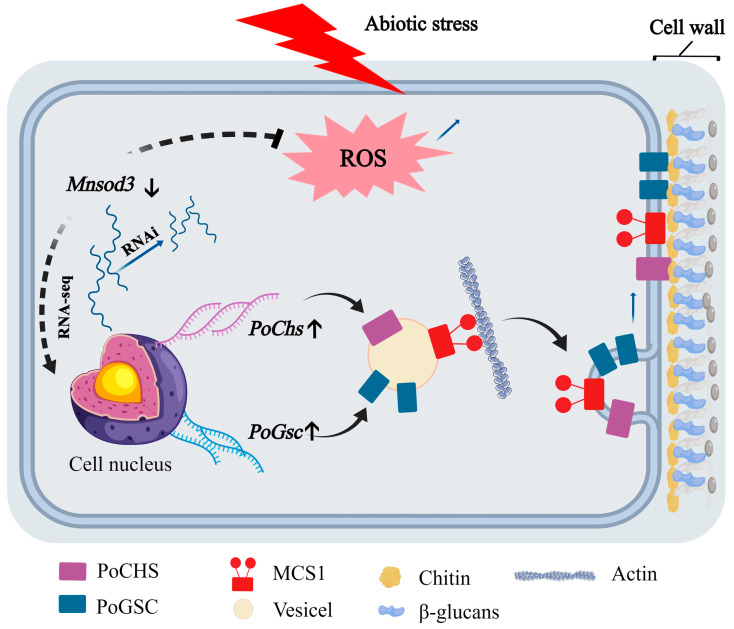
Schematic diagram of the regulatory mechanism of *Mnsod3* in response to abiotic stress during the growth and development of *P. ostreatus*.

In summary, this study revealed that *Mnsod3* plays an important role in the growth and development of *P. ostreatus*. During the process of nutritional growth, RNAi of *Mnsod3* enhances the heat tolerance of mycelia and promotes their recoverable growth after heat stress. During the formation of primordia, the RNAi of *Mnsod3* enhances the tolerance of mycelia to cold and light stress and inhibits the formation of primordia. This phenomenon may be caused by the regulation of the cell wall by the RNAi of *Mnsod3*. Figure 8 shows that under abiotic stress, the RNAi of *Mnsod3* is accompanied by increased expression of the CHS and GSC encoding gene families and increased cell wall synthesis. These research results not only clarify the role of *Mnsod3* in the growth and development of edible fungi but also provide targets for the targeted breeding of edible fungi. In future work, we will further explore the upstream regulatory factors and their mechanisms of action of MnSOD. In future work, we will further explore the upstream regulatory factors and their mechanisms of action of MnSOD.

## Data Availability

The original contributions presented in this study are included in the article/Supplementary Material. Further inquiries can be directed to the corresponding authors.

## Supplementary Materials

The following supporting information can be downloaded at: https://www.mdpi.com/article/10.3390/jof12010048/s1, Figure S1. Energy generation during the growth and development of *P. ostreatus*. (**A**) NADH content at different developmental stages. (**B**) The ratio of NAD^+^/NADH at different developmental stages. (**C**) ATP content at different developmental stages. (**D**) NADH content in different parts of the fruiting body. (**E**) The ratio of NAD^+^/NADH in different parts of the fruiting body. (**F**) ATP content in different parts of the fruiting body. Different letters indicate significant differences among the samples (*p* < 0.05 according to Duncan’s test). Figure S2. The addition of exogenous DDC regulates the growth and development of *P. ostreatus*. (**A**) Primordia. (**B**) Fruiting body. (**C**) Cap color. Figure S3. Effect of DDC on the expression level of the *Mnsod* gene family. (**A**) *Mnsod1.* (**B**) *Mnsod2*. (**C**) *Mnsod3*. Different letters indicate significant differences among the samples (*p* < 0.05 according to Duncan’s test). Figure S4. Effects of H_2_O_2_ on energy metabolism. (**A**) H_2_O_2_ content. (**B**) CAT activity. (**C**) NADH content. (**D**) The ratio of NAD^+^/NADH. (**E**) ATP content. Different letters indicate significant differences among the samples (*p* < 0.05 according to Duncan’s test). Table S1. Primers used in this study. Table S2. Key DEGs may play important roles in the ability of RNAi-*Mnsod3* to increase mycelial heat tolerance.
