# Supplementary material for: The Interference of Mnsod3 Enhances the Tolerance of Pleurotus ostreatus Mycelia to Abiotic Stress by Reshaping the Cell Wall
Source: J Fungi (Basel). 2026 Jan 10;12(1):48. doi: 10.3390/jof12010048 (PMC12843154; doi:10.3390/jof12010048)
Supplement: Supplementary file 1 [file jof-12-00048-s001.zip › jof-4055839-supplementary.pdf]

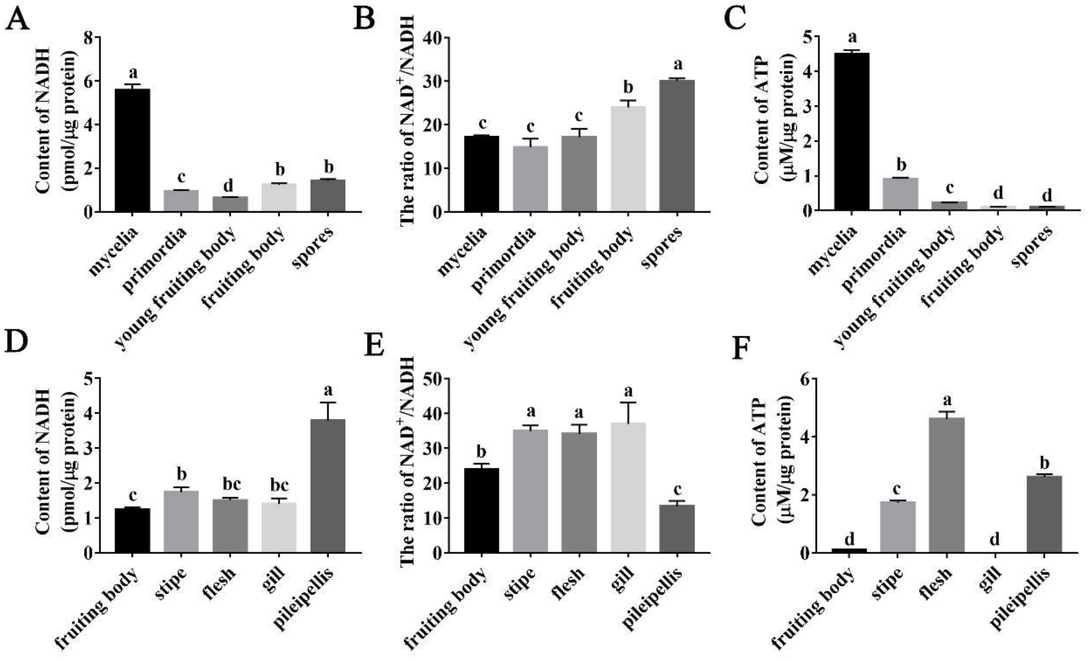

Figure S1 Energy generation during the growth and development of *P. ostreatus*. (A) NADH content at different developmental stages. (B) The ratio of NAD^+/NADH at different developmental stages. (C) ATP content at different developmental stages. (D) NADH content in different parts of the fruiting body. (E) The ratio of NAD^+/NADH in different parts of the fruiting body. (F) ATP content in different parts of the fruiting body. Different letters indicate significant differences among the samples (P<0.05 according to Duncan's test).

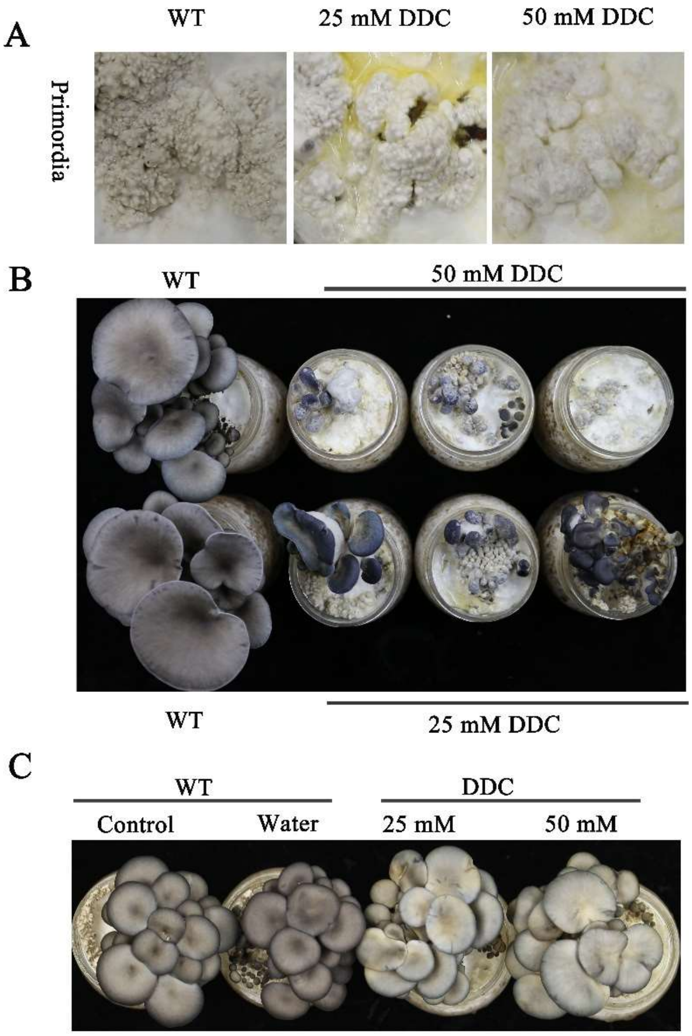

Figure S2 The addition of exogenous DDC regulates the growth and development of *P. ostreatus*. (A) Primordia. (B) Fruiting body. (C) Cap color.

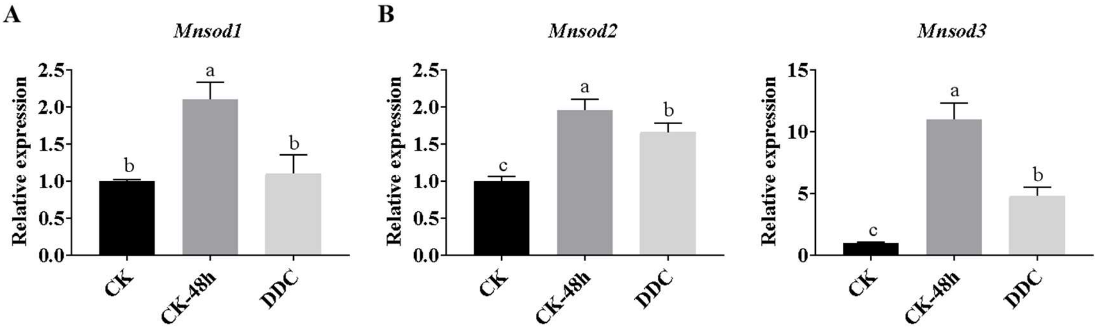

Figure S3 Effect of DDC on the expression level of *Mnsod* gene family. (A) *Mnsod1*. (B) *Mnsod2*. (C) *Mnsod3*. Different letters indicate significant differences among the samples (P<0.05 according to Duncan's test).

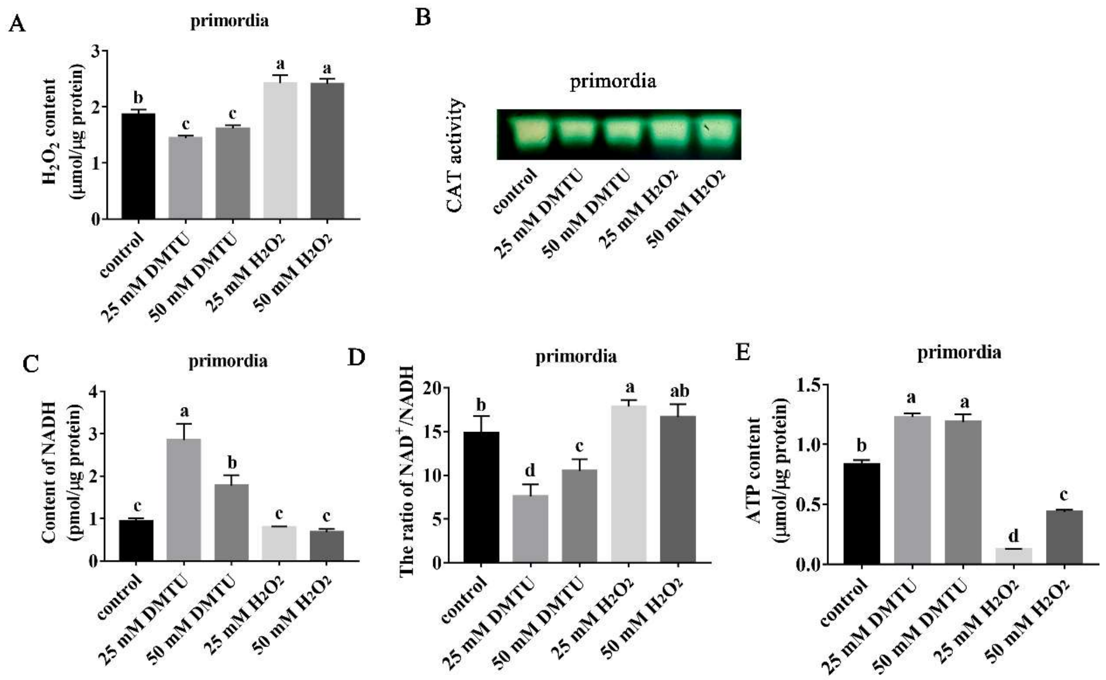

Figure S4 Effects of H_2O_2 on energy metabolism. (A) H_2O_2 content. (B) CAT activity. (C) NADH content. (D) The ratio of NAD^+/NADH. (E) ATP content. Different letters indicate significant differences among the samples (*P*<0.05 according to Duncan's test).

Table S1 Primers used in this study.

Primer	Sequence (5′→3′)	Note
PE-F	TCGCGGATCCGAATTATGAATGGCCTCCGTTTAGC	Construction of PE plasmid
PE-R	TGCTCGAGTGCGGCCCTAAACGTTTCGCATCTTGGTCC	
RNAi-*sence*-F	CCATCTCCTCAGATCATGAATGGCCTCCGTTTAGC	
RNAi-*sence*-R	TAAGCTCTAAACTAGCGTAGGGGCATCGAAATCG	
RNAi-*anti*-F	TGCCCCTACGACTAGCAGGCCTTGGTCCGGGTT	Construction of OE and
RNAi-*anti*-R	CAATTCTAGAGGGCCATGAATGGCCTCCGTTTAGC	RNAi plasmids
OE_F	GGTCAAAGTTACTAGATGAATGGCCTCCGTTTAGC	
OE_R	CAATTCTAGAGGGCCCTAAACGTTTCGCATCTTGGTCC	
hyg-F	CGACAGATCCGGTCGGCATCTACTCTATTTCTT	Detection of transformants
hyg-R	TCTCGTGCTTTCAGCTTCGATGTAGGAGGG	
qPCR_*Mnsod1*-F	ACACGAAGCATCATCAGACCTA	
qPCR_*Mnsod1*-R	GAAGAGCGAGTGGTTGATATGG	Detection of qPCR
qPCR_*Mnsod2*-F	GTTCTCCTAGCAGCGAAGA	
qPCR_*Mnsod2*-R	CATTCCCCGTTTTAAGTGAC	
qPCR_*Mnsod3*-F	TTGAACGAGACTTTGGCACC	
qPCR_*Mnsod3*-R	ATGATCGGCGCGTGAGTTATC	
qPCR_*β-actin*-F	GCGATGAACAATAGCAGGG	Reference
qPCR_*β-actin*-R	GCTGGTATCCACGAGACAAC	

Table S2 Key DEGs may play important roles in the ability of RNAi-Mnsod3 to increase mycelial heat tolerance.

Gene ID	GO ID	Description	PFAM
G10264	GO:0009277	Fungal-type cell wall	Fungal hydrophobin
G13205	GO:0009277	Fungal-type cell wall	Fungal hydrophobin
G7753	GO:0009277	Fungal-type cell wall	Fungal hydrophobin
G6727	GO:0071555	Description： cell wall organization	-
G8398	GO:0051118	Glucan endo-1,3-α-glucosidase activity	WSC domain
G8101	GO:0016020	Membrane	Glycosyl hydrolase family 47
G10008	GO:0016021	Integral component of membrane	-
G10486	GO:0016021	Integral component of membrane	Flavin-binding monooxygenase-like
G10941	GO:0016021	Integral component of membrane	Fatty acid desaturase
G11168	GO:0016021	Integral component of membrane	-
G11236	GO:0016021	Integral component of membrane	Major Facilitator Superfamily
G11414	GO:0016021	Integral component of membrane	F-box-like
G11984	GO:0016021	Integral component of membrane	Protein of unknown function
G12174	GO:0016021	Integral component of membrane	Membrane-associating domain
G13117	GO:0016021	Integral component of membrane	-
G2212	GO:0016021	Integral component of membrane	-
G3396	GO:0016021	Integral component of membrane	-
G3911	GO:0016021	Integral component of membrane	-
G7441	GO:0016021	Integral component of membrane	-
G7509	GO:0016021	Integral component of membrane	CFEM domain
G8723	GO:0016021	Integral component of membrane	SNARE associated Golgi protein
G8998	GO:0016021	Integral component of membrane	-
G8999	GO:0016021	Integral component of membrane	-
G9817	GO:0035673	Oligopeptide transmembrane transporter activity	OPT oligopeptide transporter protein
G1439	GO:0034599	Cellular response to oxidative stress	Peroxidase
G12127	GO:0004784	Superoxide dismutase activity	Iron/manganese superoxide dismutases
